# Neonatal Mortality Risk Associated with Preterm Birth in East Africa, Adjusted by Weight for Gestational Age: Individual Participant Level Meta-Analysis

**DOI:** 10.1371/journal.pmed.1001292

**Published:** 2012-08-14

**Authors:** Tanya Marchant, Barbara Willey, Joanne Katz, Siân Clarke, Simon Kariuki, Feiko ter Kuile, John Lusingu, Richard Ndyomugyenyi, Christentze Schmiegelow, Deborah Watson-Jones, Joanna Armstrong Schellenberg

**Affiliations:** 1 Faculty of Infectious and Tropical Diseases, London School of Hygiene & Tropical Medicine, London, United Kingdom; 2 Malaria Centre, London School of Hygiene & Tropical Medicine, London, United Kingdom; 3 Maternal Reproductive and Child Health Centre (MARCH), London School of Hygiene & Tropical Medicine, London, United Kingdom; 4 Faculty of Public Health and Policy, London School of Hygiene & Tropical Medicine, London, United Kingdom; 5 Department of International Health, Program in Global Disease Epidemiology and Control, Johns Hopkins Bloomberg School of Public Health, Baltimore, Maryland, United States of America; 6 Kenya Medical Research Institute/Centre for Global Health Research, Kisumu, Kenya; 7 Centers for Disease Control and Prevention Kenya, Kisumu, Kenya; 8 Child and Reproductive Health Group, Liverpool School of Tropical Medicine, Liverpool, United Kingdom; 9 National Institute Medical Research, Tanga, Tanzania; 10 Vector Control Division, Ministry of Health, Uganda; 11 Centre for Medical Parasitology, Department of International Health, Immunology and Microbiology, University of Copenhagen, Copenhagen, Denmark; 12 Department of Infectious Diseases Copenhagen University Hospital (Rigshospitalet), Copenhagen, Denmark; 13 Mwanza Intervention Trials Unit, National Institute for Medical Research, Mwanza, Tanzania; Aga Khan University, Pakistan

## Abstract

**Background:**

Low birth weight and prematurity are amongst the strongest predictors of neonatal death. However, the extent to which they act independently is poorly understood. Our objective was to estimate the neonatal mortality risk associated with preterm birth when stratified by weight for gestational age in the high mortality setting of East Africa.

**Methods and Findings:**

Members and collaborators of the Malaria and the MARCH Centers, at the London School of Hygiene & Tropical Medicine, were contacted and protocols reviewed for East African studies that measured (1) birth weight, (2) gestational age at birth using antenatal ultrasound or neonatal assessment, and (3) neonatal mortality. Ten datasets were identified and four met the inclusion criteria. The four datasets (from Uganda, Kenya, and two from Tanzania) contained 5,727 births recorded between 1999–2010. 4,843 births had complete outcome data and were included in an individual participant level meta-analysis. 99% of 445 low birth weight (<2,500 g) babies were either preterm (<37 weeks gestation) or small for gestational age (below tenth percentile of weight for gestational age). 52% of 87 neonatal deaths occurred in preterm or small for gestational age babies. Babies born <34 weeks gestation had the highest odds of death compared to term babies (odds ratio [OR] 58.7 [95% CI 28.4–121.4]), with little difference when stratified by weight for gestational age. Babies born 34–36 weeks gestation with appropriate weight for gestational age had just three times the likelihood of neonatal death compared to babies born term, (OR 3.2 [95% CI 1.0–10.7]), but the likelihood for babies born 34–36 weeks who were also small for gestational age was 20 times higher (OR 19.8 [95% CI 8.3–47.4]). Only 1% of babies were born moderately premature and small for gestational age, but this group suffered 8% of deaths. Individual level data on newborns are scarce in East Africa; potential biases arising due to the non-systematic selection of the individual studies, or due to the methods applied for estimating gestational age, are discussed.

**Conclusions:**

Moderately preterm babies who are also small for gestational age experience a considerably increased likelihood of neonatal death in East Africa.

*Please see later in the article for the Editors' Summary.*

## Abstract 2

In an analysis of four datasets from East Africa, Tanya Marchant and colleagues investigate the neonatal mortality risk associated with preterm birth and how this changes with weight for gestational age.

## Editors' Summary

**Background:**

Worldwide, every year around 3.3 million babies die within their first month of life and the proportion of under-five child deaths that are now in the neonatal period (the first 28 days of life) has increased in all regions of the world and is currently estimated at 41%. Of these deaths, over 90% occur in low- and middle-income countries, and a third of all neonatal deaths occur in sub-Saharan Africa. Low birth weight (defined as <2,500 g) is one of the biggest risk factors associated with neonatal deaths but it is the causes of low birth weight, rather than the low weight itself that is thought to lead to neonatal deaths. The two main causes of low birth weight are preterm birth (delivery before 37 weeks gestation) and/or restricted growth in the womb (intra-uterine growth retardation), resulting in babies who are small for their dates (defined as being in the lowest 10% of weight expected for gestational age with reference to a US population).

**Why Was This Study Done?:**

Despite growing international attention focused on neonatal mortality in recent years, the relative importance of low birth weight, small for gestational age, and preterm birth in causing newborn deaths remains unclear. So in this study, the researchers investigated these relationships by calculating the risk of neonatal mortality associated with preterm birth after adjusting for weight for gestational age by conducting a meta-analysis (synthesis of the data) using information from studies reporting neonatal mortality conducted in sub-Saharan Africa.

**What Did the Researchers Do and Find?:**

The researchers identified potential African datasets and selected four out of a possible ten to include in their analysis as these studies included three essential birth outcomes: birth weight; gestational age measured using antenatal ultrasound, or neonatal assessment on the day of birth; and neonatal mortality. These four studies were conducted in Kenya, Tanzania, and Uganda, all in East Africa. The researchers analysed each study separately but also conducted a pooled statistical analysis on all four studies. To give a more detailed analysis, the researchers categorized babies into six groups taking into account whether the babies were moderately preterm (born at 34–36 weeks) or very preterm (born before 34 weeks) and whether their weight was appropriate for their gestational age.

The researchers included a total of 4,843 live births in their analysis and found that overall, 9.2% of babies were low birth weight, 4.0% were preterm, and 20.4% were small for gestational age. Amongst low birth weight babies, 26.1% were preterm, 85.0% were small for gestational age, and 98.8% were either preterm or small for gestational age. In their detailed analysis, the researchers found that the odds (chance) of death in the first 28 days of life were seven times higher for babies born low birth weight compared to those with normal birth weight, with low birth weight infants experiencing a neonatal mortality rate of 80.9/1,000 live births. The odds of death were twice as high for babies born small for gestational age compared to those born appropriate for gestational age, giving a neonatal mortality rate of 29.3/1,000 live births. Furthermore, compared to those born at term, the odds of death were over six times higher for babies born moderately preterm and almost 60 times higher for babies born very preterm with almost half of all very preterm babies dying in the first 28 days of life, giving a neonatal mortality rate 473.6/1,000 live births. However, moderately preterm babies who were small for gestational age had a much greater odds of death than moderately preterm babies who were of the appropriate weight for their gestational age.

**What Do These Findings Mean?:**

These findings from East Africa show that babies born either small for gestational age or preterm contributed 52% of neonatal deaths. The detailed analysis suggests that babies born preterm are at the greatest risk of death, but size for gestational age also plays an important role especially in moderately preterm babies. The results from this study emphasize the pressing need to find ways to prevent preterm delivery and intra-uterine growth retardation and also illustrate the importance of measuring and reporting outcomes of individual babies.

**Additional Information:**

Please access these Web sites via the online version of this summary at http://dx.doi.org/10.1371/journal.pmed.1001292.

- A recent *PLOS Medicine* study by Oestergaard et al. has the latest global figures on neonatal mortality
- UNICEF provides information on neonatal mortality
- The World Health Organization (WHO) provides factsheets on the causes of neonatal mortality, including preterm birth

## Introduction

Low birth weight (<2,500 g) is one of the strongest predictors of neonatal mortality (death in the first 28 d of life), is routinely collected and reported in health literature, and is associated with a complex set of fetal and maternal characteristics [1]. However low birth weight itself is a consequence of either preterm birth (<37 completed weeks gestation), or intra-uterine growth restriction resulting in small for gestational age births (defined as being below the tenth percentile of weight for gestational age of a U.S.-based reference population) [2], or a combination of the two: low birth weight per se is not thought to be on the causal pathway to neonatal mortality [3]–[6].

There is an absence of data to explain the way in which low birth weight, small for gestational age, and preterm risks interact with neonatal mortality in high mortality burden settings. In the United States, where the neonatal mortality rate (NMR) is relatively low at 5/1,000 live births [4], mortality outcomes are reported to vary across groups of weight for gestational age [7],[8], with newborns born small for gestational age at 34–36 wk gestation estimated to have a neonatal mortality risk as much as 44 times higher than the risk experienced by newborns born with appropriate weight for gestational age and at term [2],[9],[10].

In contrast, the NMR in sub-Saharan Africa is persistently high at an estimated 41/1,000 live births, translating to around 1.2 million deaths in 2008, which represents around one-third of all global neonatal deaths [4],[11]. Yet there is remarkably little individual level data available, despite recent attention from policy makers [6]. Using mortality models and vital registration statistics where they exist, it has been estimated that around 28% of newborns died because of complications arising from preterm birth [11], and around 80% of those who died, 970,000 newborns, would have been born with low birth weight [4],[12]. However, there is a lack of empirical data with which to examine the relative importance of low birth weight, small for gestational age, and preterm birth in causing newborn deaths.

In a meta-analysis of individual level data collected across East Africa, we estimated the odds of neonatal mortality associated with preterm birth after adjusting for weight for gestational age. We accessed data from studies over a 10-y period that collected high quality birth weight and gestational age data, and which reported newborn survival to at least 28 d of life.

## Methods

### Ethics Statement

Approval was obtained from the following: (1) Institutional Review Board, Kenya Medical Research Institute, and US Centers for Disease Control and Prevention; (2) National Medical Research Coordinating Committee (Tanzania), and London School of Hygiene & Tropical Medicine; (3) The Uganda National Council for Science and Technology, and London School of Hygiene & Tropical Medicine; (4) National Medical Research Coordinating Committee (Tanzania).

The methodology was developed in partnership with colleagues at the Child Health Epidemiology Reference Group (http://cherg.org/).

### Eligibility Criteria

To be eligible for inclusion in this analysis a minimum of three birth outcome measures were required. First, birth weight defined as weight measured within 72 h of birth using a calibrated scale. Second, gestational age defined either using antenatal ultrasound, or neonatal assessment using the Dubowitz [13] or Ballard scale [14] on the day of birth. Third, neonatal mortality required a protocol for active follow-up to at least the 28th day of life.

A set of maternal explanatory variables was also defined, and included maternal education level (as a proxy for socio-economic status), whether the birth took place at home or in a health facility, and parity of the mother. The availability of maternal malaria infection indicators was explored across all datasets but was not a criterion for selection.

### Search Strategy

The search for eligible datasets was conducted October–December 2010 by TM, JAS, and BW. To our knowledge, no studies have been designed or published from Africa with the specific aim of answering our research question, thus the sampling strategy was convenience, not systematic, taking two stages. First we contacted all members of the Malaria Center, and of the Maternal Reproductive and Child Health Center (MARCH) at the London School of Hygiene & Tropical Medicine (LSHTM) to enquire about eligible African datasets, also extending the enquiry to their international collaborators. Second, we reviewed protocols of studies identified to ensure that our eligibility criteria had been met.

A total of 10 possible African datasets were identified, four of which were defined as eligible and are described below. These four were all from East Africa, were completed within an 11-y interval (1999–2010), included all newborn outcome and maternal explanatory variables, and measured peripheral maternal malaria infection at delivery. Six datasets did not meet our criteria for gestational age or birth weight (two from Ghana, one from each of Tanzania, Sudan, Zambia, Malawi).

### Studies Assembled

A summary of methods used by each study when measuring newborn outcomes is provided in Table 1. A summary of the newborn outcomes for all newborns measured is presented in Table 2, irrespective of whether the newborn had all three outcome measures (as defined by the “included” population).

**Table 1:** Study methods for measurement of newborn outcomes (birth weight, gestational age at birth. and mortality follow-up).

Outcome Measure	Study Methods
**Birth weight**	
Kenya: Asembo Bay	Digital scale measuring to nearest 10 g
Tanzania: Mwanza	Hospital digital scale, or project digital scale for home births, both measuring to nearest 10 g
Uganda: Kabale	Digital scale measuring to the nearest 10 g
Tanzania: Korogwe	Digital strain gauge scale to nearest 10 g or a spring scale to nearest 50 g
**Gestational age at birth**	
Kenya: Asembo Bay	Neonatal assessment (Ballard [14]) at birth
Tanzania: Mwanza	Antenatal trans-abdominal ultrasound, and neonatal assessment (Dubowitz [13]) at birth)
Uganda: Kabale	Neonatal assessment (Ballard [[14])
Tanzania: Korogwe	Antenatal trans-abdominal ultrasound
**Mortality follow-up**	
Kenya: Asembo Bay	Active community follow-up to at least 1 y of life (home visits at 0, 7, 14, 28 d of life, and every 2 wk thereafter)
Tanzania: Mwanza	Active community surveillance to 28 d of life by project staff
Uganda: Kabale	Active community follow-up to 28 d of life (home visits at 0, 7, 14, 21, 28 d of life)
Tanzania: Korogwe	Active community follow-up and surveillance of clinic attendance to at least 30 d of life by project staff

**Table 2:** Prevalence of outcome measures (neonatal mortality, low birth weight, and prematurity) amongst all measured babies in each study.

Outcome	Korogwe, Tanzania	Mwanza, Tanzania	Asembo Bay, Kenya	Kabale, Uganda
**Total number of live births**	915	1,496	1,828	1,488
**Neonatal mortality**				
Number measured	863	1,492	1,813	1,478
Outcome for all measured	NMR 29.0/1,000	NMR 22.1/1,000	NMR 20.4/1,000	NMR 16.9/1,000
**Low birth weight (<2,500 g)**				
Number measured	819	1,243	1,459	1,487
Outcome for all measured	10.9% (8.7–13.0)	10.2% (8.5–11.9)	10.6% (9.1–12.1)	7.0 (5.7–8.3)
**Preterm (<37 wk)**				
Number measured	910	1,238	1,656	1,488
Outcome for all measured	5.1% (3.6–6.5)	2.7% (1.8–3.6)	3.1% (2.2–3.9)	5.9 (4.7–7.0)

### The Asembo Bay Cohort Project

The Asembo Bay Cohort Project in Siaya District, western Kenya (completed 1999) [15] was conducted within the context of a large community-based group randomized controlled trial designed to assess the impact of insecticide treated nets (ITNs) on mortality in children less than 5 y of age.

#### Study population

19 villages were included for longitudinal follow-up of pregnant women and their newborns and these cohort observations are included here. A monthly census identified pregnant women who were recruited and prospectively followed to birth and for at least 1 y thereafter.

#### Included observations

Of the 1,828 live births recorded, 1,465 (80%) were included in this analysis. Because of the community-based design of the study, the data represent rural infants born at home and in the local clinics. Newborn measures not taken within 72 h of birth were excluded from our analysis. The likely effect was to miss very early deaths and therefore underestimate the incidence of adverse outcomes. The excluded population had a higher NMR 51.7 per 1,000 compared to 13 per 1,000 in those included (X^2^ *p*<0.001), and had a higher prevalence of preterm birth (X^2^ *p*<0.02) (Table 3).

**Table 3:** Distribution of study characteristics for infants included (survival follow-up to 28 d and birth weight and gestational age data available) and for infants excluded (at least one data point missing of survival to 28 d, birth weight, and gestational age).

Study Characteristics	Korogwe, Tanzania		Mwanza, Tanzania		Asembo Bay, Kenya		Kabale, Uganda		All Observations	
	Included *n* = 731	Excluded *n* = 184	Included *n* = 1,170	Excluded *n* = 326	Included *n* = 1,465	Excluded *n* = 363	Included *n* = 1,477	Excluded *n* = 11	Included *n* = 4,843	Excluded *n* = 884
**Newborn outcomes**										
NRM^a^	32.8	7.6	16.2	43.5^b^	13.0	51.7^b^	16.9	N/A	18.0	41.1^b^
Mean birth weight g (SD)	3,100 (517)	3,243 (581)	3,091 (456)	3,015 (635)	3,068 (455)	3,270 (679)^b^	3,192 (477)	2,877 (748)^b^	3,116 (475)	3,198 (653)^b^
Mean gestation wk (SD)	39.2 (1.8)	39.3 (1.6)	39.4 (1.4)	39.1 (1.3)	39 (1.3)	38.6(1.4)	37.3 (0.8)	36.4(1.8)	38.7 (1.5)	38.9 (1.6)^b^
Low birth weight %	11.3	7.3	9.6	18.4^b^	9.9	10.2	6.9	10.0	9.2	10.9
Preterm %	4.9	5.6	2.7	1.4	2.8	5.7^b^	5.7	18.1	4.0	5.3
Small for gestational age %^c^	21.9	20.4	25.3	28.5	26.4	11.4^b^	9.9	20.0	20.4	16.3
Female %	50.5	47.3	47.8	53.0	51.4	45.5^b^	48.8	45.4	49.6	48.7
Twins %	3.8	2.1	0	0	0	0	2.1	18.1^b^	1.2	0.7
**Delivery characteristics** d										
Vaginal deliveries %	90.0	92.3	94.0	99.0^b^	100	96.0^b^	91.9	100	94.9	96.7
Facility deliveries %	88.5	52.5^b^	98.8	10.1^b^	8.2	14.2^b^	100	100	70.3	24.0^b^
**Maternal characteristics** d										
Mean age in years (SD)	26.9 (6.2)	27.1 (6.1)	23.9 (6.1)	25.2 (7.7)^b^	25.7 (6.8)	25.5 (6.7)	25.1 (5.7)	24.3 (5.7)	25.3 (6.3)	25.7 (7.0)
First live birth %	23.9	19.1	32.9	19.0^b^	N/A	N/A	37.5	18.1	11.6	11.1
No education %	6.4	5.9	11.0	18.4^b^	10.8	11.2	10.3	9.0	10.0	12.8^b^
Positive for malaria %^e^	1.6	1.5	18.0	15.7	30.3	29.6	15.4	20.0	18.5	18.9

a NRM expressed per 1,000 live births.

b indicates difference in distribution between included and excluded for individual projects, or all combined, to be significant at the 5% level.

c Using Alexander definition <10%.

d Both included and excluded newborn groups had missing observations from the mother.

e Malaria parasites of any Plasmodium species in the maternal peripheral blood at delivery detected by blood slide in Kenya (Asembo Bay), Tanzania (Mwanza), and Uganda (Kabale), and by rapid diagnostic test in Tanzania (Korogwe).

### The Antenatal Syphilis Screening and Treatment Project

The Antenatal Syphilis Screening and Treatment project in Mwanza, northwest Tanzania (completed 2000) [16] examined whether single-dose benzathine penicillin treatment was adequate to prevent adverse pregnancy outcomes.

#### Study population

Syphilis screening was carried out for all women attending the antenatal clinic. Eligible antenatal attendees with syphilis (defined by positive rapid plasma reagin test [RPR]), followed by the next two eligible antenatal attendees without syphilis, were recruited. Hospital and home deliveries were followed up actively, and for at least 28 d thereafter.

#### Included observations

Of the 1,496 live births in the cohort, 1,170 (78%) were included in this analysis. The mortality rate of those excluded (43.5/1,000) was almost double that of infants included (16.2/1,000) in the analysis (X^2^ *p*<0.03). Compared with included babies, excluded babies were more frequently born at home, had a higher prevalence of low birth weight (X^2^ *p*<0.007), and were born to less educated mothers (X^2^ *p*<0.001, Table 3).

### The Ugandan Malaria Study

The Ugandan malaria study (the efficacy and cost effectiveness of malaria prevention in pregnancy in an area of low and unstable transmission in Kabale District) (completed 2006) [17], was an individually randomized three-arm intervention trial examining the efficacy of intermittent preventive treatment in pregnancy with sulphadoxine-pyrimethamine (SP) in pregnancy (IPTp-SP) when combined with ITNs, compared to IPTp-SP alone, or to ITNs alone.

#### Study population

Women attending antenatal services for the first time that pregnancy, and who were estimated to be ≤28 wk gestation, were enrolled and examined. Women with severe anemia (Hb<70 g/l), or other severe disease, were excluded and referred for treatment, and those who developed severe anemia during follow-up were withdrawn from the study and treated. All women were visited at home 1 wk after enrolment at antenatal clinic, at approximately 36 wk gestation, and at term. Women who subsequently delivered at home were visited within 7 d after birth and are not included in this analysis.

#### Included observations

Of the 5,226 women monitored to delivery, only 1,602 delivered in a health facility where birth weight and gestational age assessment was carried out within 72 h. Of these, there were 1,488 live births, 1.477 (99%) of whom had complete data and are included in this analysis. The excluded babies born in health facilities had more twin births (X^2^ *p*<0.001) and had lower mean birth weight (*t*-test *p* = 0.03); the NMR amongst included infants was 16.9/1,000 and was not available for those excluded (Table 3).

### The Strategies to Prevent Pregnancy Associated Malaria Project

The Strategies to Prevent Pregnancy Associated Malaria project (STOPPAM; www.stoppam.org) [18] in Korogwe, Tanzania (completed 2010), was a prospective cohort study of pregnant women to quantify the effects of pregnancy associated malaria and identify a vaccine candidate.

#### Study population

Antenatal care attendees with a gestational age of 24 wk or less based on ultrasound evaluation, and who planned to give birth at Korogwe District Hospital were enrolled. All hospital deliveries were attended by STOPPAM staff on a 24-h rota, and home deliveries were monitored through home visits by project staff.

#### Included observations

Of the 915 live births in the cohort, 731 (80%) had complete information for this analysis. There were more home births amongst excluded infants (X^2^ *p*<0.001). One quarter of the excluded births (46/184) did not have mortality follow-up to the 28th day of life; the NMR amongst those included was 32.8/1,000 but only 7.6/1,000 amongst those excluded, although this difference did not reach statistical significance (X^2^ *p*<0.11) (Table 3).

### Statistical Methods

NMR was calculated as observed deaths within 28 d divided by observed live births. Low birth weight was defined as weight less than 2,500 g. Gestational age at birth was categorized as: term (≥37 completed weeks of gestation), moderately preterm (34–36 wk), very preterm (<34 wk). Small weight for gestational age was defined as being below the tenth percentile of weight for gestation of a US-based reference population [19]. Babies were further categorized into six groups: (1) appropriate for gestational age and term; (2) appropriate for gestational age and 34–36 wk gestation; (3) appropriate for gestational age and <34 wk gestation; (4) small for gestational age and term; (5) small for gestational age and 34–36 wk gestation; (6) small for gestational age and <34 wk gestation.

Taken together the four eligible studies provide strength to answer this research question, but were individually small and no previous attempt had been made to conduct this analysis. Odds ratios (ORs) and corresponding 95% CIs for neonatal mortality with relation to the exposures of low birth weight (compared to not low birth weight), moderate or very preterm (compared to term), small for gestational age (compared to appropriate for gestational age), and weight for gestational age stratified by preterm (compared to appropriate weight for gestational age and term), were calculated in each of the four datasets individually, using logistic regression. Individual measures of effect and uncertainty measures from the four studies were then included in fixed effects meta-analyses, using the metan commands in STATA. Study estimates were combined using inverse variance weighting, and pooled ORs were summarised with Mantel-Haenszel methods. Between study heterogeneity was investigated using the *I* ^2^ statistic [20] and CIs around the *l* ^2^ statistic were calculated using the user generated i2ci.ado command in STATA [21].

To inform whether ORs included within meta-analyses should be crude or adjusted, sub-group investigation for confounding of the relationship with neonatal mortality was carried out within each of the four included studies using available covariates (sex, twin, delivery characteristics, and maternal characteristics including malaria positivity at delivery).

Additionally, in order to investigate the influence of exclusions due to incomplete data, sensitivity analyses comparing characteristics of included and excluded infants were conducted. Continuous variables were compared using *t*-tests, and proportions using Chi-squared tests. All analyses were carried out using STATA version 11 (Stata Corporation).

The proportional distribution of newborn deaths for preterm stratified by weight for gestational age was calculated. Finally, we estimated the proportion of neonatal deaths associated with risk factors [(overall NMR - NMR for babies without the risk factor)/overall NMR], and the mortality that could be attributed to different weight for gestational age and preterm outcomes, i.e. the attributable risk percent [1-(NMR amongst babies without the risk factor/NMR amongst babies with the risk factor)]. Both of these estimates rest on the assumption that avoiding the risk factors preterm birth or low birth weight percentile for gestational age would not affect any other factor.

## Results

### Completeness of Data

**Figure 1:**
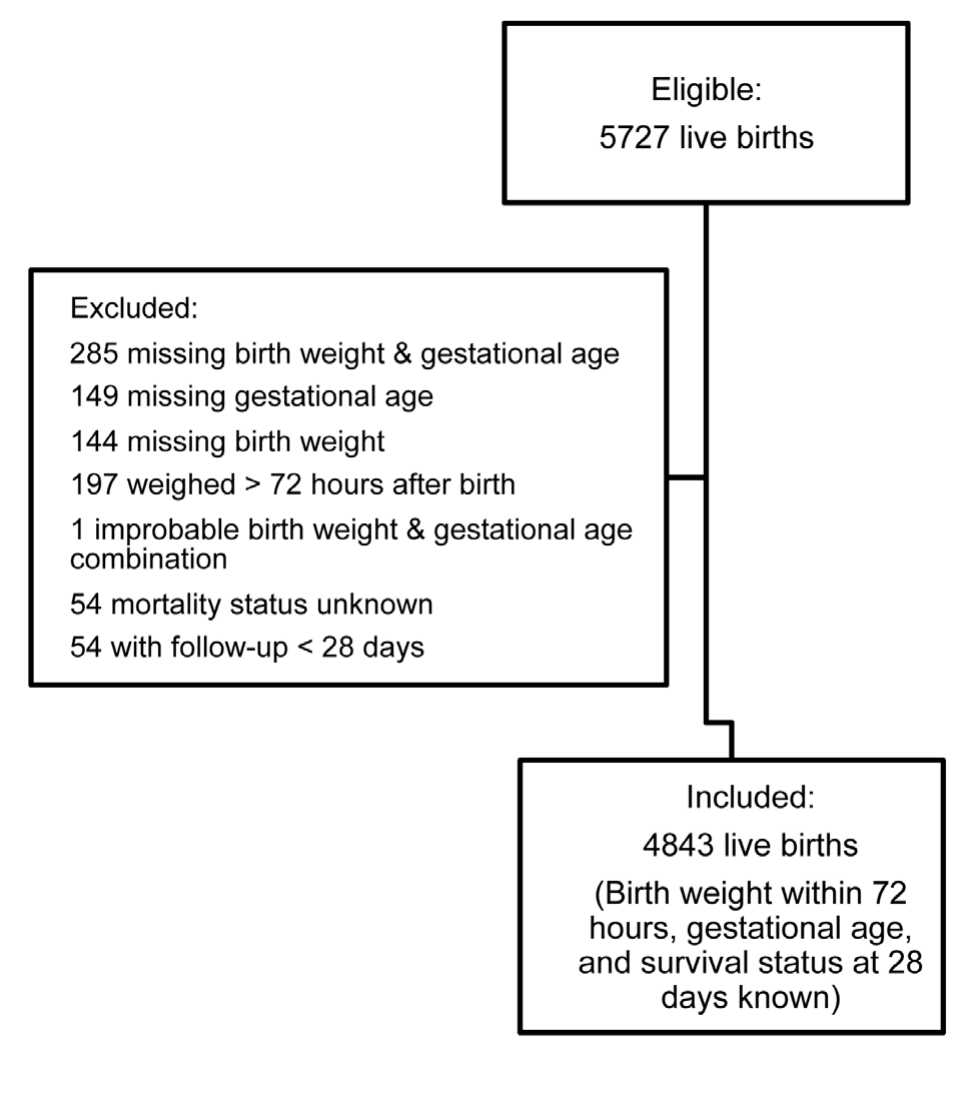
Flow chart of combined study population.

From these four studies 5,727 live births were observed, of whom 4,843 (85%) had a complete set of outcome data and were included in the analysis (Figure 1). The mortality estimate for all those included (18.0 per 1,000 live births [95% CI 14.2–21.7]) was less than half that of all those excluded (41.1 per 1,000 live births [95% CI 27.3–54.9]), indicating a downward bias in point estimates of neonatal mortality rates (Table 3).

### Mortality Outcomes by Low Birth Weight, Small for Gestational Age, and Preterm

In the individual studies, between 6.9% and 11.3% of babies were low birth weight, being 9.2% (95% CI 8.4–10.0) for all studies combined. Between 2.7% and 5.7% of babies were preterm, being 4.0% (95% CI 3.5–4.6) for all studies combined. Between 9.9% and 26.4% of babies were small for gestational age, being 20.4% (95% CI 19.3–21.6) for all studies combined (Table 3). Amongst low birth weight babies, 26.1% (95% CI 22.0–30.4) were preterm, 85.0% (95% CI 81.7–88.5) were small for gestational age, and 98.8% (95% CI 97.3–99.6) were either preterm or small for gestational age.

**Figure 2:**
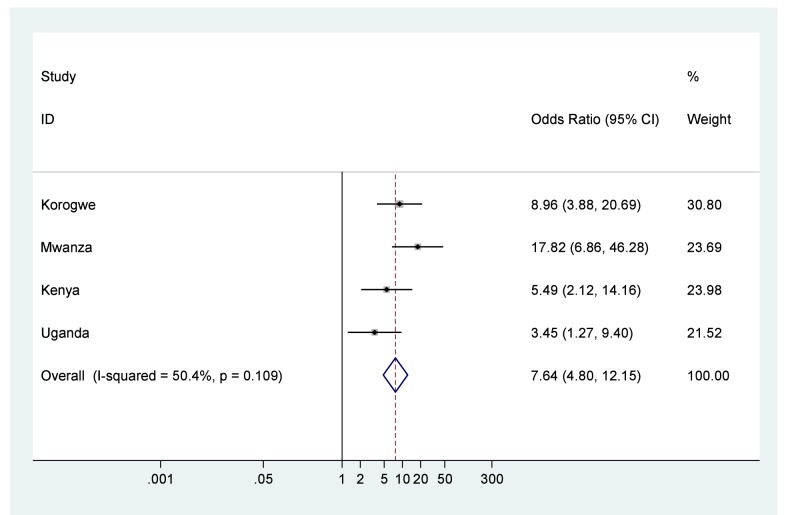
Neonatal mortality outcomes for babies with birth weight <2,500 g compared to babies with birth weight ≥2,500 g. Note: 95% CI for *I* ^2^ was 0%–83.6%.

**Figure 3:**
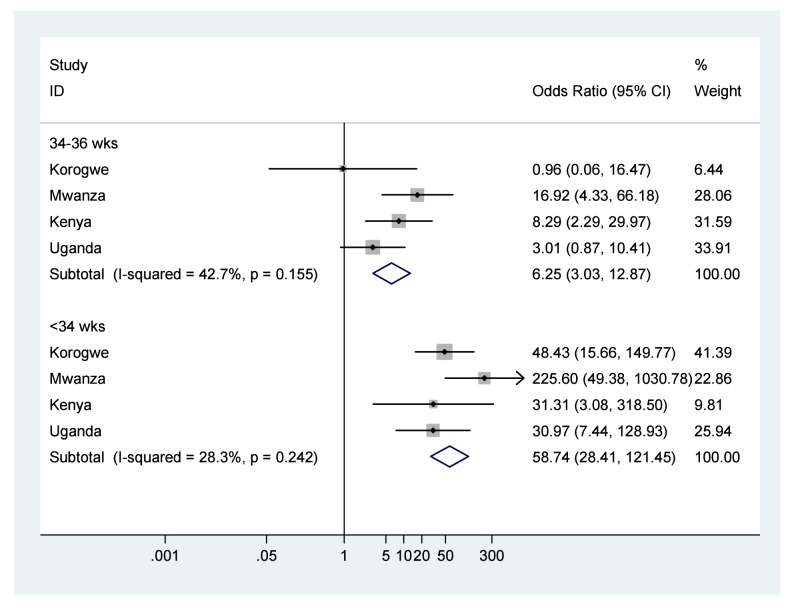
Neonatal mortality outcomes for babies born moderately (34–36 wk) or very (<34 wk) preterm compared to babies born at term ≥37 wk. Note: 95% CI for *I* ^2^ 34–36 wk was 0%–80.8%, and for *I* ^2^<34 wk was 0%–73.4%.

**Figure 4:**
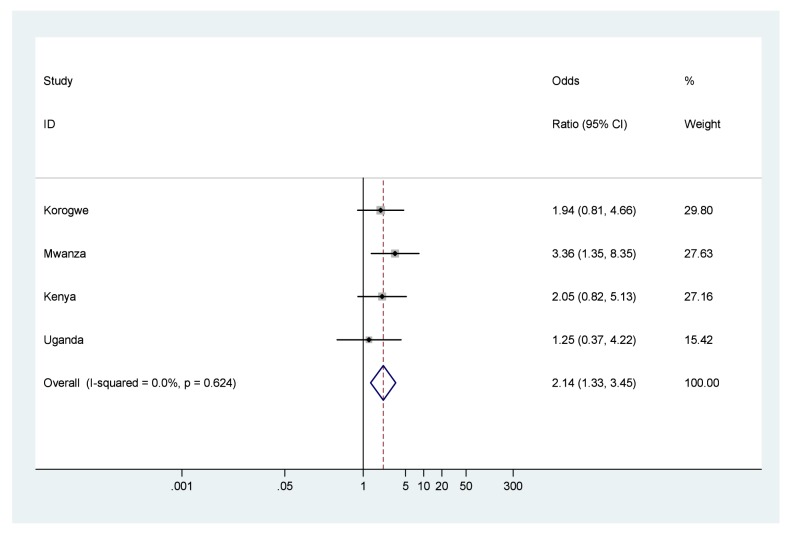
Neonatal mortality outcomes for babies born small for gestational age (<10%) compared to babies born appropriate for gestational age. Note: 95% CI for *I* ^2^ was 0%–73.9%.

**Figure 5:**
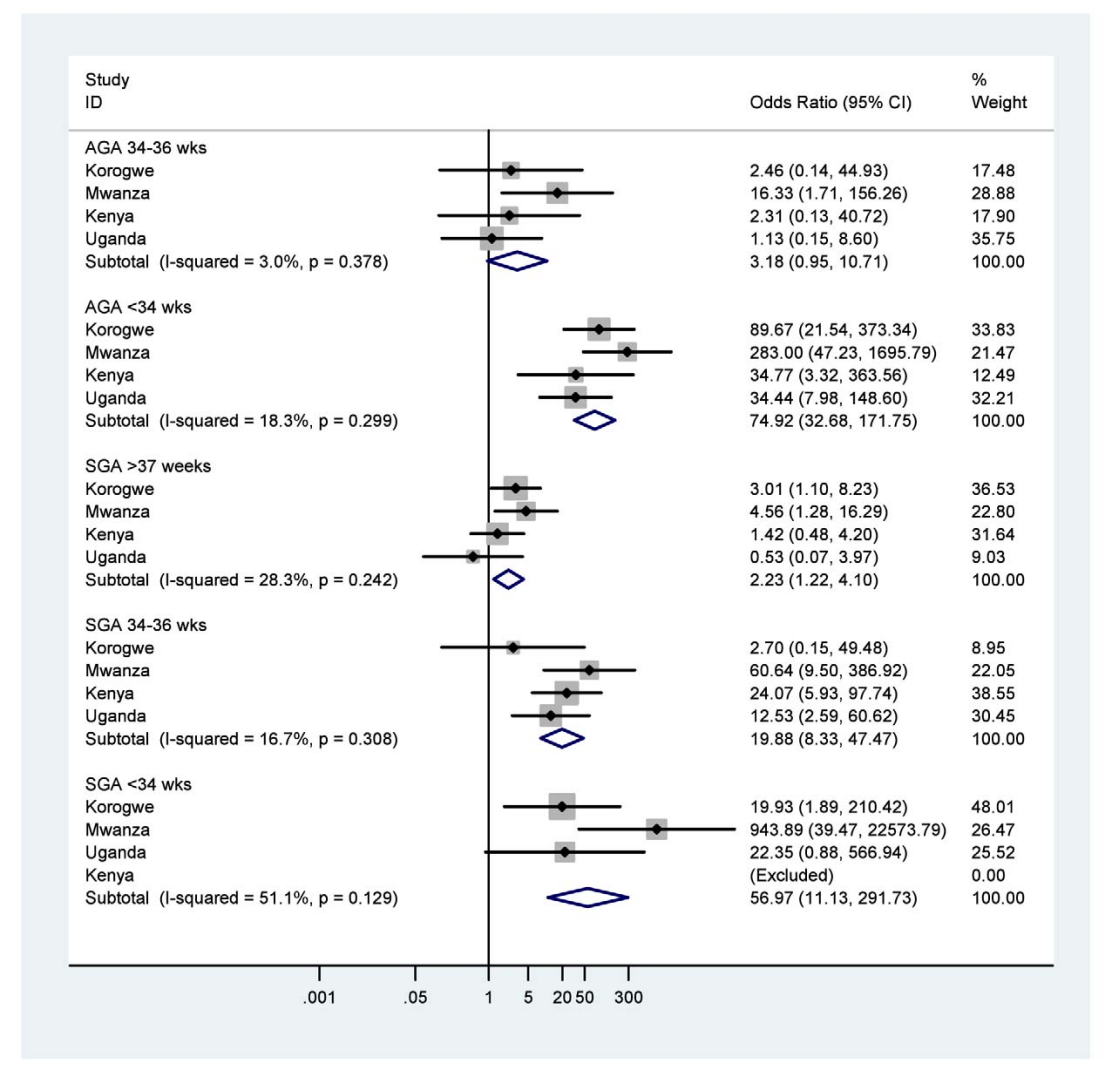
Neonatal mortality outcomes for very or moderately preterm babies (<34 or 34–36 wk), stratified by weight for gestational age (appropriate [AGA]≥10%, or small [SGA] <10%), using term and appropriate for gestational age as the reference group. Note: 95% CI for *I* ^2^ was 0%–85.1% for AGA 34–36 wk 0%–87.5% for AGA<34 wk, 0%–73.4% for SGA>36 wk, 0%–87.3% for SGA 34–36 wk, and 0%–85.9% for SGA<34 wk. There were no newborns SGA<34 wk in Kenya (shown as Excluded).

Forest plots of results of meta-analyses showing ORs and 95% CIs for each study, and the pooled measure of effect and corresponding 95% CIs, are shown in Figure 2 for low birth weight, Figure 3 for preterm birth, and Figure 4 for small for gestational age. Figure 5 shows the results of neonatal mortality for preterm, stratified by weight for gestational age. All models include study-specific crude ORs, as no evidence of confounding was found for available covariates. Fixed effects meta-analysis models are presented due to the relatively low heterogeneity, as summarised by the *I* ^2^ values (although it should be noted that with only four studies there was very low power to detect heterogeneity and the 95% CIs around the *l* ^2^ values range from 0% (no heterogeneity) to 87% (high heterogeneity) [21].

The odds of death in the first 28 d of life were seven times higher for babies born low birth weight compared to those with normal birth weight (OR 7.6, 95% CI 4.8–12.1), and low birth weight infants experienced a NRM of 80.9/1,000 live births (Figure 2; Table 4). The odds of death were over six times higher for babies born moderately preterm compared to those born term (OR 6.2, 95% CI 3.0–12.8), and almost 60 times higher for babies born very preterm compared to those born term (OR 58.7, 95% CI 28.4–121.4), with almost half of very preterm babies dying in the first 28 d of life, NRM 473.6/1,000 live births (Figure 3; Table 4). The odds of death were twice as high for babies born small for gestational age compared to those born appropriate for gestational age (OR 2.1, 95% CI 1.3–3.5), NRM 29.3/1,000 live births (Figure 4; Table 4).

**Table 4:** Meta-analysis of neonatal mortality outcomes by birth weight, gestational age, weight for gestational age, and stratified by weight for gestational age.

Outcome	Live Births	Deaths	Mortality Rate^a^	95% CI	OR^b^		Distribution of Births	Distribution of Deaths	Attributable Risk Percent^c^
					OR	95% CI			
**All**	4,843	87	18.0	14.2–21.7	—	—	100%	100%	—
**Birth weight**									
≥2,500 g	4,398	51	11.6	8.4–14.8	Ref	—	90.9	58.6	Ref
<2,500 g	445	36	80.9	55.4–106.3	7.6	4.80–12.2	9.2	41.3	85.7
**Gestational age**									
≥37 wk	4,649	60	12.9	9.7–16.1	Ref	—	96.1	68.9	Ref
34–36 wk	156	9	57.7	20.7–94.7	6.2	3.0–12.8	3.2	10.3	77.6
<34 wk	38	18	473.6	307.3–640	58.7	28.4–121.4	0.8	20.7	97.3
**Weight for gestational age**									
Appropriate for gestational age	3,854	57	15.0	11.0–18.6	Ref	—	79.6	66.3	Ref
Small for gestational age	989	29	29.3	18.8–39.9	2.1	1.3–3.5	20.5	33.7	49.5
**Stratified by weight for gestational age**									
*Appropriate for gestational age*									
≥37 wk	3,718	42	11.0	7.7–14.4	Ref	—	76.7	48.3	Ref
34–36 wk	106	2	18.8	−7.3 to 44.3	3.18	1.0–10.7	2.2	2.3	41.5
<34 wk	30	14	466.7	277.2–656.1	74.9	32.6–171.7	0.6	16.1	97.6
*Small for gestational age*									
≥37 wk	934	19	20.3	11.3–29.4	2.23	1.2–4.10	19.3	21.8	45.8
34–36 wk	48	7	145.8	42.2–249.4	19.88	8.3–47.5	1.0	8.0	92.5
<34 wk	7	3	428.6	−65.8 to 922.9	56.97	11.1–291.7	0.1	3.4	97.4

a NRM expressed per 1,000 live births.

b Calculated from fixed effects meta-analysis, using Mantel-Haenszel methods for pooled ORs.

c Attributable risk percent calculated as the observed mortality that could be attributed to different weight for gestational age outcomes.

Using babies born with weight appropriate for gestational age and at term as reference, the odds for neonatal mortality were three times higher for those born appropriate for gestational age at 34–36 wk (OR 3.2, 95% CI 0.9–10.7; NRM 18.8/1,000 live births), but 75 times higher for those born appropriate for gestational age at <34 wk (OR 74.9, 95% CI 32.6–171.7: NRM 466.7/1,000 live births) (Figure 5; Table 4). Again using babies born appropriate for gestational age and term as reference, the odds for mortality were doubled for babies born small for gestational age at term (OR 2.2, 95% CI 1.2–4.1; NRM 20.3/1,000 live births), were 20 times greater for babies born small for gestational age at 34–36 wk (OR 19.9, 95% CI 8.3–47.4; NRM 145.8/1,000 live births), and were 57 times greater for babies born small for gestational age at <34 wk gestation (OR 57.0, 95% CI 11.1–291.7; NRM 428.6/1,000 live births).

After stratifying by method of assessing gestational age, ultrasound or neonatal assessment, the direction of these findings remains consistent, although the CIs are wide (Table 5). In particular, the finding of elevated odds for neonatal mortality amongst babies born small for gestational age at 34–36 wk persists (OR 24.6, 95% CI 5.1–117.8 for those measured by ultrasound; OR 18.0, 95% CI 6.3–51.4 for those measured by neonatal assessment).

**Table 5:** Methodological stratification of neonatal mortality outcomes for preterm babies (<34 and 34–36 wk), stratified by weight for gestational age (appropriate [AGA]≥10%, or small [SGA]<10%), using term and appropriate for gestational age as the reference group.

Gestational Age Estimation Method	OR	95% CI
**Ultrasound** a		
*Appropriate for gestational age*		
≥37 wk	Ref	—
34–36 wk	8.0	1.3–47.5
<34 wk	140.1	45.9–427.5
*Small for gestational age*		
≥37 wk	3.5	1.6–7.7
34–36 wk	24.6	5.1–117.8
<34 wk	78.5	11.8–520.9
**Neonatal assessment** b		
*Appropriate for gestational age*		
≥37 wk	Ref	—
34–36 wk	1.4	0.2–7.5
<34 wk	34.5	9.9–119.4
*Small for gestational age*		
≥37 wk	1.1	0.4–2.9
34–36 wk	18.0	6.3–51.4
<34 wk (Uganda only)	22.3	0.8–566.9

a Mwanza and Korogwe, Tanzania.

b Uganda and Kenya.

### Contribution of Preterm and Small Weight for Gestational Age to Neonatal Mortality

23% (1,125/4,843) of the live births, but 53% (45/87) of the newborn deaths were amongst newborns born either small for gestational age or preterm. Less than 1% (37/4,843) of live births, but 20% (17/87) of deaths, were amongst very preterm infants (<34 wk). Just 1% (48/4,843) of live births, but 8% (7/87) of deaths, were amongst those born moderately preterm (34–36 wk) and small for gestational age.

Overall, 28% of neonatal mortality was associated with being born preterm [(18 – 12.9)/18 * 100], and 39% of neonatal mortality was associated with being born either preterm or small for gestational age [(18.0−11.0)/18 * 100], assuming that all babies would have the same risk of neonatal death if they were born term and appropriate for gestational age (Table 4).

98% of the mortality risk (the attributable risk percent) of babies born appropriate for gestational age at <34 wk was attributed to them having been born very preterm [(466.7−11.0)/466.7 * 100]. Over 90% of the neonatal mortality risk of all small for gestational age and preterm babies (<37 wk) was attributed to them being born small for gestational age and preterm (Table 4).

## Discussion

99% of low birth weight babies were either small for gestational age or preterm. Just 23% of babies were born either small for gestational age or preterm but they contributed 52% of the neonatal deaths. The 4% of babies who were born preterm were at highest likelihood of death, accounting for 30% of the neonatal deaths, with over 90% of their mortality risk being attributed to being preterm. However this analysis of data from East Africa revealed that weight for gestational age played an important role for moderately preterm babies. The odds of neonatal mortality of babies born 34–36 wk gestation and appropriate weight for gestational age was just three times higher than term babies of appropriate weight, but was 20 times higher amongst babies born 34–36 wk gestation and small for gestational age.

Preterm birth is a direct cause of mortality but also aggravates the effect of other risk factors; small for gestational age may arise because of intra-uterine growth retardation which has been shown to increase the risk of mortality and morbidity [6],[22],[23]. Therefore being small for gestational age (especially if that was due to intra-uterine growth retardation) and preterm (even if only moderately so) may synergistically lead to the increased odds observed here. These findings have public health importance when thinking about the potential of interventions that focus on reducing intra-uterine growth retardation, or on reducing prematurity in this setting. Malaria in pregnancy interventions, for example, may have a marked impact to reduce the occurrence of severe neonatal outcomes but push a larger number of newborns into moderate categories of risk.

Previous studies have reported the mortality risk associated with separate measures of birth outcome in East Africa [24], but to our knowledge, this analysis of preterm births stratified by weight for gestational age has not been presented before for an African population and thus provides much needed evidence relevant to priority setting in a high mortality setting [25]. One limitation was that the search for studies was not systematic because early discussions and searches of the literature did not reveal any studies that had addressed this problem in the African setting. The analysis does not attempt to present population level estimates of low birth weight, small for gestational age, preterm, or neonatal mortality, but rather to disentangle the relationship between them when they occur. A particular strength has been the access to detailed newborn datasets from a relatively homogenous geographical spread, and the rigorous definitions applied to measures of birth weight and gestational age.

Nonetheless, there are three inter-related limitations in this study, each a reflection of the difficulty of obtaining high quality individual level birth outcome data in this setting. First, there was selection bias in that 70% of the included babies were born in a health facility, compared to only 24% of excluded babies, and an expected 50% at the population level for East Africa; thus the included mothers may be better health seekers than those excluded. These study findings may be an underestimate of the true population level effects.

The second limitation, consistent with the first, was the exclusion of around 15% of live births because of missing data for birth weight or gestational age information, or survival to 28 d of life. In our sensitivity analysis there was evidence of bias in that, for three of the four studies, neonatal mortality of those excluded was far higher than of those included, as was the prevalence of preterm birth. The most likely explanation for this finding is that some very early deaths were excluded or classified as lost to follow-up because babies died before birth weight or gestational age could be estimated (32% of those excluded [285/884] had missing birth weight and gestational age data, 17% [149/884] had missing gestational age, and 16% [144/884] had missing birth weight). Again, this limitation may have led to underestimation of the mortality risks, especially in the first days of life, associated with low birth weight, preterm, and small for gestational age. Imputation was considered as an approach to address the problem of missing data, a key factor being to have gestational age where birth weight was missing. However, given the quantity of missing gestational age data we did not feel confident that there were enough good covariates with which to make a sensible prediction model.

Finally, there may be measurement error for gestational age (and therefore classification of size for gestational age) because of the methods used to determine gestational age, and there may also be misclassification of size for gestational age because of the use of US-based reference population for standardised birth weight by gestational age values [2]. On the later point, currently there is a lack of standardised birth weight by gestational age values for sub-Saharan Africa: one multi-country study (www.intergrowth21.org.uk) that aims to address this is on-going and results are expected to be available in 2014. Some data from individual studies exist, for example in 2011 a study in Botswana developed standard values there and found that Botswana-born preterm infants had higher average birth weights than US-born infants [26]. Similar conclusions were also reported from Congo [27], and it has previously been suggested that such findings may be due to different growth velocity at the end of pregnancy for some groups [28]. If the findings from the Botswana and Congo studies are generalisable for Tanzania, Kenya, and Uganda, then using the US standard could have led us to underestimate small for gestational age amongst preterm babies. However, the authors of those studies noted that the accurate dating of gestation was problematic, [26] or may have represented an atypically healthy population. [27]

Indeed, gestational age data across Africa are scarce, and what data exist are prone to bias—most markedly for preterm newborns who are at highest mortality risk [8],[29],[30]. Of the three available gestation dating methods, neonatal assessments have consistently been shown to underestimate very preterm infants by as much as 2 wk compared to ultrasound, date of last monthly period has been shown to overestimate prematurity and to be susceptible to serious reporting errors, while ultrasound is generally considered to be the most precise dating method but is rarely available in sub-Saharan Africa [31]–[33]. In our study, only 4% of the newborns included in the analysis were defined as preterm. A previous meta-analysis had estimated preterm births in East Africa to be amongst the highest in the world at 14% [34], but over half the included studies in that analysis did not report the method of estimation and the findings should be interpreted with caution.

Reflecting on the implications of these uncertainties for the principal findings, we observed an increase in odds of neonatal mortality to be consistently larger when gestational age was assessed by ultrasound compared to neonatal assessment, but both methods show results in the same direction, and the lower limit of confidence around the estimates was very close for both (Table 5). We also observed a consistent pattern across countries. As such, we have confidence that the finding of an increased odds of neonatal mortality amongst those born moderately premature and small for gestational age (SGA) in comparison to those born moderately premature and appropriate for gestational age (AGA) in East Africa is secure. However, because of the challenges of gathering high quality population-level newborn data in the East African setting, especially gestational age and classification of size for gestational age, we cannot be certain about the true magnitude of that increase. Given the growing emphasis on the prevention of newborn deaths across sub-Saharan Africa, the measurement and reporting of individual newborn outcomes should be given greater emphasis.

Three issues particularly exacerbate interventions to prevent preterm and small births in the East African setting: (1) the aetiology of small for gestational age and preterm birth is multi-factorial [6],[35]; (2) around half of babies are born at home and experience higher mortality risks than those born in facilities [36]; and (3) small for gestational age and preterm babies born at home are frequently not identified as needing extra care [37]. As the deadline for achieving Millennium Development Goals grows near, implementing newborn interventions that target small for gestational age as well as preterm birth, and are adaptable to poorly resourced health facility or community settings is vital [38]–[40].

### Conclusion

Preterm or small for gestation births accounted for 52% of newborn deaths in this analysis of data from East Africa. Preterm birth had the strongest association with death, but there was also an additional risk for moderately preterm babies born small for gestational age compared to those born moderately preterm and appropriate for gestational age. 8% of babies who died were born moderately preterm and small for gestational age: if this was extrapolated to the estimated 1.2 million neonatal deaths in sub-Saharan Africa in 2008 this finding would translate to 96,000 African newborns lost.

## Abbreviations

NMR: neonatal mortality rate
OR: odds ratio
